# Reaching the ‘first 90’: Gaps in coverage of HIV testing among people living with HIV in 16 African countries

**DOI:** 10.1371/journal.pone.0186316

**Published:** 2017-10-12

**Authors:** Sarah Staveteig, Trevor N. Croft, Kathryn T. Kampa, Sara K. Head

**Affiliations:** 1 Avenir Health, Glastonbury, Connecticut, United States of America; 2 The Demographic and Health Surveys (DHS) Program, Rockville, Maryland, United States of America; 3 International Health and Development Division, ICF, Rockville, Maryland, United States of America; 4 Health, Research, Informatics & Technology Division, ICF, Atlanta, Georgia, United States of America; 5 Independent Researcher, Washington, DC, United States of America; University of Washington, UNITED STATES

## Abstract

**Background:**

UNAIDS has recently proposed a set of three ambitious targets that, if achieved, are predicted to end the AIDS epidemic by 2030. The targets, known as 90-90-90, call for 90% of people living with HIV (PLHIV) to know their status, 90% of PLHIV to receive antiretroviral therapy, and 90% of those on antiretroviral therapy to achieve viral suppression by the year 2020. We examine the first of these targets, focusing on sub-Saharan Africa, the region of the world most affected by HIV, to measure the proportion of PLHIV estimated to know their HIV status, and to identify background and behavioral characteristics significantly associated with gaps in ever testing among PLHIV.

**Methods and findings:**

We analyze cross-sectional population-based data from the Demographic and Health Surveys (DHS) and AIDS Indicator Surveys (AIS) fielded since 2010 in 16 sub-Saharan African countries where voluntary serological testing was recently conducted: Burkina Faso, Cameroon, Chad, Cote d’Ivoire, Ethiopia, Gabon, Lesotho, Malawi, Namibia, Rwanda, Sierra Leone, Tanzania, Togo, Uganda, Zambia, and Zimbabwe. Survey response rates averaged 95.0% (range 89.3–99.5%), while consent to serotesting averaged 94.9% (range 88.7–99.6%).

This study, which includes more than 14,000 respondents living with HIV, finds that 69% of PLHIV in the average study country have ever been tested for HIV (range 34–95%). Based on timing of the last test and on ART coverage, we estimate that 54% of PLHIV in the average country are aware of their status (range 26–84%).

Adjusted logistic regression finds that men (median adjusted odds ratio [AOR] = 0.38), adults with less than primary education (median AOR = 0.31), and adolescents (median AOR = 0.32) are consistently less likely to have ever been tested for HIV than women, adults with secondary and above education, and adults age 30–39, respectively. In most countries unadjusted logistic regression also finds significant gaps in testing among the poorest groups and those reporting never having had sex.

**Conclusion:**

The fact that an average of 54% of PLHIV in these 16 countries are estimated to know their status reflects encouraging progress. However, not only is this average far short of the 90% target set by UNAIDS for 2020, but it also implies that in the average study country nearly one-half of PLHIV are unable to access lifesaving care and treatment because they are unaware that they are HIV-positive.

Several gaps in HIV testing coverage exist, particularly among adolescents, the least educated, and men. While the need to target demographic groups at greatest risk of HIV continues, additional interventions focused on reaching men and on reaching socially vulnerable populations such as adolescents, the poorest, and the least educated are essential.

## Introduction

In 2016, the Joint United Nations Programme on HIV/AIDS (UNAIDS) estimated 1.9 million new HIV infections annually among individuals age 15 and older. Of the estimated 36.7 million [34.0–39.8 million] people living with HIV (PLHIV) worldwide, 25.5 million [23.0–28.3 million] are in sub-Saharan Africa, which globally is the region hardest hit by the AIDS epidemic [1]. Deaths among adolescents living with HIV have been increasing since 1990, and HIV/AIDS is now the leading cause of death among adolescents in sub-Saharan Africa [2].

Growing momentum in the global commitment to ending the AIDS epidemic led to the establishment of the 90-90-90 targets by UNAIDS in 2014. This set of ambitious targets proposes that, by 2020, 90% of PLHIV will know their status, 90% of people diagnosed with HIV will receive antiretroviral therapy (ART), and 90% of people receiving ART will have achieved viral suppression. UNAIDS predicts that reaching these targets by 2020 will enable the world to end the AIDS epidemic by 2030, creating profound health and economic benefits [3]. This study examines the first target, with a focus on HIV testing uptake and estimated knowledge of HIV status among PLHIV in 16 sub-Saharan African countries—Burkina Faso, Cameroon, Chad, Cote d’Ivoire, Ethiopia, Gabon, Lesotho, Malawi, Namibia, Rwanda, Sierra Leone, Tanzania, Togo, Uganda, Zambia, and Zimbabwe—and the factors associated with uptake among this population, by country.

HIV testing coverage has made notable progress over the past decade. In Africa the proportion of people with HIV who are aware of their HIV-positive status grew from an estimated 10% in 2005 to 55% in 2015 [4]. This achievement becomes even more significant when considering that individuals who know their status are less likely to engage in HIV-related risk behaviors [5] and, following an HIV-positive diagnosis, to have early initiation of ART, as recommended by the World Health Organization (WHO), which can lead to a suppressed viral load, thereby reducing the risk of HIV transmission [3].

However, HIV testing access and uptake is not uniform across countries or communities in sub-Saharan Africa, and substantial gaps in coverage remain. While recent estimates report that 77% of all people diagnosed with HIV are on ART, 40% of all people with HIV remain undiagnosed [4]. Although the number of annual HIV tests has increased, many people at greatest risk remain unreached by testing [4]. Studies conducted in sub-Saharan Africa have identified several factors that may influence an individual’s decision to seek or participate in HIV testing; these often include sex, residence, and education level [6–10]. Factors such as antenatal care visits and other maternal health services provided for pregnant women, distance to an HIV testing facility, cost and convenience, knowledge and understanding of HIV and treatment, previous testing experience, number of lifetime sexual partners, and age have also been found to influence HIV testing behaviors [6–10].

In an effort to increase HIV testing and treatment, several initiatives have been implemented in the 16 countries studied, with varying degrees of success. One approach has been to integrate prevention of mother-to-child transmission (PMTCT) programs with maternal and child health services. Rwanda, for example, began adopting a more family-centered approach in 2003. The approach placed strong emphasis on counseling and encouragement of HIV testing, as well as system-strengthening measures such as increasing the number of personnel at clinics when ANC services are being provided, streamlining patient flows, cutting wait times, and assuring confidentiality. Uptake of HIV testing and PMTCT services among pregnant women rose to above 90% at the 18 clinics that implemented the new approaches, and, by 2009, more than 80% of male partners were also taking HIV tests, compared with 16% in 2003 [11].

Large-scale testing campaigns have helped increase uptake among people who do not typically use healthcare services; this strategy has been particularly common in East African countries. Often, these campaigns use community-based testing, which is effective in reaching large numbers of first-time testers and diagnosing PLHIV at earlier stages of the infection. In Uganda, community health campaigns aim to achieve universal testing across a community by removing significant barriers and offering rapid HIV testing. These campaigns offer HIV testing within broader service delivery, such as hypertension screening for adults and deworming of young children, thereby normalizing HIV testing as a part of routine health care. Testing locations are also decentralized to minimize travel costs and waiting time, and community members are encouraged to attend, regardless of their perceived HIV risk [12].

To extend testing coverage to groups with limited access to existing services, new delivery models and revised national policies have been developed. Many new models put an emphasis on delivering services to rural areas and hard-to-reach groups, using various mobile approaches and community facilities [11]. Additionally, many adolescents living with HIV have been left behind due to barriers related to national HIV testing age-of-consent laws, which act as key barrier to uptake of services among young people [13]. WHO addressed the special needs of adolescents in the 2015 treatment guideline revisions by including specific recommendations regarding when to start ART and service delivery approaches for adolescents (age 10–19). The guidelines emphasize adolescent-friendly health services within HIV settings to increase engagement with adolescents, improve outcomes, and promote the integration of services for sexually transmitted infections and family planning into HIV care settings [14].

Overall, countries have been working with international partners to scale-up testing and treatment services rapidly in an effort to reach the 90-90-90 goals, but the success of these individual initiatives rely on a broader imperative to invest in strengthening and expanding health systems, infrastructure, and national policies. In light of the ‘first 90’ target and the broader context of HIV testing, this study seeks to answer two questions. First, what proportion of PLHIV in the 16 study countries are estimated to know their HIV status? Second, what background and behavioral characteristics are significantly associated with gaps in ever testing among PLHIV?

## Methods

### Data

Demographic and Health Surveys (DHS) and AIDS Indicator Surveys (AIS) are nationally representative, cross-sectional surveys conducted in many countries worldwide. They are primarily funded by the U.S. Agency for International Development (USAID) and implemented with technical assistance from The DHS Program at ICF, a global consulting and technology services provider. Surveys conduct both individual adult and household interviews, and gather a number of biomarkers about health and wellbeing.

This study covers countries in sub-Saharan Africa with a DHS or AIS survey conducted in 2010 or after that included HIV serological testing. We include only the most recent survey from a country if that survey included at least 50 men and 50 women who were classified as HIV-positive, according to the survey blood test, regardless of the overall HIV prevalence measured in the survey. Sixteen countries had surveys that qualified for inclusion. The ICF Institutional Review Board, which requires compliance with the United States Department of Health and Human Services regulations for the protection of human subjects (45 CFR 46), approved the questionnaires and procedures for all surveys included in this study.

### Measures

#### Prior HIV testing

In standard DHS and AIS surveys, respondents who say they have heard of HIV are asked, *“I don’t want to know the results*, *but have you ever been tested for HIV*?*”* In earlier surveys, the term “AIDS virus” was used in place of HIV. If the respondent answers yes, they are asked a few subsequent questions—timing, place of test, and, in some cases, whether testing was voluntary or required—and then asked *“I don’t want to know the results*, *but did you get the results of the test*?*”* [15, 16].

In two surveys—Uganda 2011 and Namibia 2013—the preface *“I don’t want to know the results”* was not used, as respondents were asked about the results. In these two surveys, if respondents answered yes, after intermediary questions on timing and location of the test they were asked “*Did you get the results of the test*?*”*

Additionally, in the DHS and AIS surveys, respondents to the woman’s questionnaire who have given birth in the two years before the survey are also asked separate but similar questions about HIV testing in the context of ANC and delivery services. If they were tested at any of these points in time they are skipped out of the question on ever testing, and instead proceed to questions about timing and receipt of results.

The results of questions about ever testing and about testing during ANC and delivery described above are used to produce two key indicators in this study: ever tested and tested in the last 12 months. As is standard for these indicators, the respondent must have received the results of the last test to be considered as having been tested.

#### HIV serostatus

After questions about past testing, in some DHS and in all AIS surveys respondents are asked for consent to be anonymously tested for HIV. HIV testing undertaken during the survey process is separate from, and subsequent to, the self-reports of prior HIV testing in the questionnaire. It is possible, therefore, for respondents to be both HIV-positive and never tested for HIV; in other words, to have no knowledge of their HIV status at the time of the survey.

HIV testing protocol in DHS and AIS surveys undergoes an ethical review by the host country, by ICF International, and—for surveys receiving PEPFAR funding—by the U.S. Centers for Disease Control and Prevention (CDC). After a participant consents to be tested, interviewers or a medical staff member accompanying the interviewers collects blood drops from a finger prick on filter paper. The collection, storage, and testing of these dried blood spot samples follow strict procedures to ensure quality and reliability of the testing. The blood spots are dried overnight, packaged, and transported to a laboratory for testing. Although the standard DHS HIV testing protocol has recently changed, for most of the surveys included in this analysis, the following protocol was followed. In the laboratory, the samples were tested using an initial ELISA test, and then all positive samples and 5 to 10% of negative samples were retested with a second ELISA. For those tests with discordant results on the two ELISA tests, another test, usually a Western blot, was used to determine the result. As external quality control, all positive samples and a random sample of about 3 to 5% of the negative samples were sent to another lab not associated with the survey, and the testing protocol was repeated. The results from the independent lab were checked against the results of the main laboratory. Each filter paper has a unique bar code that can be linked to the questionnaire to allow for analysis of HIV status by respondents’ background characteristics, but the bar code does not allow the results of the HIV test to be identified with the name or location of a specific individual.

It sometimes happens that the Western blot results for a few samples are indeterminate; in such cases, the lab HIV testing algorithm used has not definitively classified a sample as positive or negative. This happens in generally five or fewer cases per country. We exclude these respondents from the numerator and denominator of our sample, on the grounds that the objectives of this analysis require individuals to have a positive or negative classification. The number of indeterminates is too small to produce reliable estimates about this group, and is too low for their exclusion to influence the overall results.

In late 2015, The DHS Program changed its algorithm for determining HIV serostatus. A confirmatory assay was added to the testing algorithm when the first two assays are positive. Results that are positive on all three assays are considered HIV-positive; results that are negative or indeterminate on the third assay are treated as HIV-negative for prevalence calculations. This affected the Malawi 2015–16 and Zimbabwe 2015 surveys: specimens that had positive results on the first two assays and a negative or indeterminate result on the third assay were classified as HIV-negative, while indeterminate results—those with discordant results on the two ELISA tests and an indeterminate result on the third test—were excluded from the numerator and denominator, as described above.

#### Estimated knowledge of HIV status

Estimating self-knowledge of HIV status is difficult, particularly among PLHIV. When HIV-positive individuals are asked directly about the results of their most recent test, the data suggest substantial underreporting [17]. For this reason, an indirect measure of self-knowledge can be an appropriate proxy.

The proxy for knowledge of HIV status among PLHIV in this analysis is based on self-reported information on prior HIV testing. Individuals who report never having been tested or having not received results of the most recent test are assumed not to know their HIV status. At the same time, individuals who were tested and received the result in the past 12 months are almost certain to know their current status. The percentage of PLHIV who are estimated to know their status, therefore, can be considered to range from a lower bound equal to the percentage of respondents who have been tested and received their test result in the past 12 months to an upper bound equal to the percentage of respondents who have ever been tested and received their test result. To add precision, the estimate can be adjusted for ART coverage among PLHIV. Specifically, ART coverage can be an alternate lower bound to testing in the past 12 months because everyone on ART can be assumed to know they are HIV-positive. If the percentage of adults on ART is higher than the percentage tested in the past 12 months, ART coverage is substituted as the low bound. The midpoint estimate is calculated to produce “*people living with HIV who are estimated to know their status*.” Our indicator is equivalent to the definition of *“people living with HIV who know their status”* introduced by UNAIDS in 2016 [18]. We prefer the addition of the word “estimated” as actual knowledge of HIV status among respondents is unobserved.

#### ART coverage

None of the 16 surveys in this analysis included a biomarker for ART use. Instead, our data source on ART coverage is the UNAIDS AIDSinfo website’s estimate of *“coverage of people receiving ART”* from the year of the survey fieldwork [19]. If survey fieldwork spanned two years, we used the estimate for ART coverage in the earlier year. Note that estimates of ART coverage are for the entire adult population (age 15 and older), while survey analysis of PLHIV pertains to adults age 15–49.

#### Covariates

The six covariates examined in this study reflect background characteristics that are associated with risk of HIV infection and with access to services, as well as behavioral characteristics that elevate the risk of HIV infection and may prompt individuals to seek testing. The covariates are sex, place of residence, age group, educational attainment, wealth tercile, and lifetime number of sexual partners.

Most covariates are self-explanatory. “Wealth tercile” is based on principal components analysis of assets, amenities, and services at the household level and divided into terciles of the household population. It is relative within surveys but not across countries; wealthier households may still be categorized as poor on an absolute basis. To create wealth terciles, we followed the procedure that The DHS Program uses to create quintiles: we weighted the household dataset by the number of household members and divided continuous scores into three equal groups, then mapped the resulting classification back to individual PLHIV. Lifetime number of sexual partners reported by respondents is divided into groups of 0, 1, 2–3, 4+, and don’t know or missing (DK/missing); the latter category includes respondents who say they have lost count and those who are unwilling to state a number. In some countries the DK/missing category is negligible, but in other countries it can be somewhat substantial and is thus kept as a separate group.

### Weighting

DHS and AIS surveys typically use a two-stage cluster sampling design to reach households and, ultimately individuals within households, as described in the DHS Sampling and Household Listing Manual [20]. Individual male and female HIV weights were applied to the data to adjust for nonresponse and to restore representativeness of the sample. Regression analysis presented in this study used complex survey commands in Stata, which adjust for sampling weights, stratification, and intra-cluster correlation.

## Results

### Response rate and HIV prevalence

Table 1 shows that across study countries the adult response rate ranged from 89.3% to 99.5% (average 95.0%). Between 88.7% and 99.6% of respondents consented to anonymous serological testing (average 94.9%). The prevalence of HIV among adults age 15–49, computed from the results of this testing, ranges from 1.0% in Burkina Faso to 24.7% in Lesotho. In total, over 14,000 PLHIV were identified through serotesting and are included in subsequent analysis.

**Table 1:** Response rate and HIV prevalence.

Survey	Survey response rate^a^ (%)	Number interviewed and eligible for serological testing^a^	Consented to testing^a^ (%)	Tested, valid results^b^	HIV prevalence^b^ (%)	Number^b^ HIV-positive
**Burkina Faso 2010**	98.1	15,025	97.6	14,607	1.0	148
**Cameroon 2011**	96.8	13,914	97.0	13,500	4.3	584
**Chad 2014–15**	95.2	10,896	94.3	10,214	1.6	163
**Cote d’Ivoire 2011–12**	91.9	9,796	88.7	8,558	3.7	317
**Ethiopia 2011**	92.0	29,383	93.5	27,254	1.5	400
**Gabon 2012**	97.4	10,640	97.7	10,444	4.1	426
**Lesotho 2014**	96.0	6,044	97.0	5,819	24.7	1,435
**Malawi 2015–16**	97.0	15,409	94.3	14,457	8.8	1,266
**Namibia 2013**	89.3	8,613	88.8	7,731	14.0	1,085
**Rwanda 2014–15**	99.5	12,362	99.6	12,302	3.0	365
**Sierra Leone 2013**	97.0	14,805	95.9	13,956	1.5	207
**Tanzania 2011–12**	92.8	19,319	92.4	17,745	5.1	908
**Togo 2013–14**	96.9	8,912	97.8	8,721	2.5	216
**Uganda 2011**	92.8	19,866	98.5	19,556	7.3	1,436
**Zambia 2013–14**	93.7	29,941	93.4	27,859	13.3	3,704
**Zimbabwe 2015**	94.2	17,973	91.3	16,141	13.8	2,229

^a^ Unweighted

^b^ Weighted with HIV sample weights; see text for details

### Estimated knowledge of HIV status

Table 2 lists three components used to calculate estimated knowledge of HIV status among PLHIV: (1) tested for HIV in the past 12 months, (2) ART coverage, and (3) ever tested for HIV, along with the resulting percentage of PLHIV estimated to know their HIV status. In Uganda, where an issue with the questionnaires prevented the surveys from accurately measuring testing in the past 12 months among all women, only the ART numbers could be used as lower bounds. Conversely, in Ethiopia data on ART coverage were not available and thus only the percentage tested in past 12 months could be used.

**Table 2:** ART coverage, testing, and estimated knowledge of HIV status among PLHIV.

Survey	Tested in past 12 months (%)	On ART^a^ (%)	Ever tested (%)	Estimated to know their HIV status^b^ (%)
**Burkina Faso 2010**	10	35	41	38
**Cameroon 2011**	31	19	68	50
**Chad 2014–15**	19	31	42	37
**Cote d’Ivoire 2011–12**	15	21	41	31
**Ethiopia 2011**	29	^d^	72	51
**Gabon 2012**	32	33	72	53
**Lesotho 2014**	49	37	85	67
**Malawi 2015–16**	39	61	89	75
**Namibia 2013**	48	61	89	75
**Rwanda 2014–15**	39	72	95	84
**Sierra Leone 2013**	9	18	34	26
**Tanzania 2011–12**	33	20	69	51
**Togo 2013–14**	18	33	61	47
**Uganda 2011**	^c^	25	69	47
**Zambia 2013–14**	44	50	82	66
**Zimbabwe 2015**	46	61	89	75

^a^ Data on antiretroviral therapy (ART) coverage is from UNAIDS estimates for adults age 15 and over. Due to the lack of decimal precision in these estimates, all other results are rounded.

^b^ Estimated to know their HIV status uses the maximum of tested in past 12 months or ART coverage as a lower bound for knowledge of HIV status and percentage ever tested as an upper bound. It is computed as the midpoint of the lower and upper bounds.

^c^ Due to a problem with the questionnaire for the Uganda 2011 AIDS Indicator Survey, data on time since last HIV test are not available for some women; therefore, these estimates are omitted.

^d^ Data listed as unavailable.

As Table 2 shows, the percentage of PLHIV tested in the past 12 months ranges from 9% in Sierra Leone to 49% in Lesotho. The percentage of PLHIV ever tested ranges from 34% in Sierra Leone to 95% in Rwanda. Among the countries studied, West African countries—Burkina Faso, Chad, Cote d’Ivoire, Sierra Leone, and Togo—tend to report the lowest percentages of recent and ever tested. Lesotho, Malawi, Namibia, Rwanda, and Zimbabwe report among the highest levels of recent and ever tested, with the level of PLHIV ever tested at or above 85% in each country. ART coverage estimated for the year of survey fieldwork ranges from 18% in Sierra Leone to 72% in Rwanda.

The percentage of PLHIV estimated to know their HIV status ranges from 26% in Sierra Leone to 84% in Rwanda. In the average country, 54% of PLHIV are aware of their status. In 5 of 16 countries, more than two-thirds of PLHIV are estimated to know their status.

### Characteristics of the study population

Table 3 shows the background characteristics of PLHIV by country. The composition of PLHIV reflects both the characteristics of the population in each country, as well as the nature of the epidemic. Women comprise the majority of adults in most countries, and they are more vulnerable than men to STIs and HIV both socially (gender inequality, violence, power) and biologically (during heterosexual transmission); therefore, we would expect PLHIV to be majority female in sub-Saharan Africa. Even so, the sex composition of PLHIV also reflects differences in risk behavior, age structure, and relative survival.

**Table 3:** percentage distribution of selected characteristics among PLHIV Age 15–49, by country.

	Burkina Faso 2010	Cameroon 2011	Chad 2014–15	Cote d’Ivoire 2011–12	Ethiopia 2011	Gabon 2012	Lesotho 2014	Malawi 2015–16	Namibia 2013	Rwanda 2014–15	Sierra Leone 2013	Tanzania 2011–12	Togo 2013–14	Uganda 2011	Zambia 2013–14	Zimbabwe 2015
**Sex**																
**Male**	34.3	31.1	37.6	34.6	31.7	25.6	34.3	34.2	37.0	33.1	38.4	33.9	31.7	36.8	40.2	35.2
**Female**	65.7	68.9	62.4	65.4	68.3	74.4	65.7	65.8	63.0	66.9	61.6	66.1	68.3	63.2	59.8	64.8
**Place of residence**																
**Urban**	56.9	60.6	70.6	60.2	66.6	88.2	42.8	31.0	53.6	41.7	56.5	37.7	65.3	24.3	63.4	38.8
**Rural**	43.1	39.4	29.4	39.8	33.4	11.8	57.2	69.0	46.4	58.3	43.5	62.3	34.7	75.7	36.6	61.2
**Age group**																
**15–19**	5.7	6.7	12.1	2.7	2.0	5.0	5.0	5.7	3.5	4.2	16.6	4.7	2.0	7.3	7.8	5.7
**20–29**	22.0	31.6	33.9	29.0	33.0	29.4	31.4	23.4	22.2	26.5	33.4	27.0	16.6	29.8	28.2	22.6
**30–39**	44.9	39.3	39.4	35.2	45.9	39.9	38.7	41.4	45.3	33.6	36.8	39.6	44.4	38.1	38.4	40.1
**40–49**	27.4	22.4	14.6	33.0	19.1	25.7	24.9	29.4	29.0	35.7	13.3	28.8	37.0	24.8	25.6	31.7
**Education**																
**None**	54.9	8.7	42.7	50.5	31.8	2.5	5.0	12.4	9.0	15.2	42.8	12.9	22.4	12.8	5.7	0.9
**Primary**	21.4	37.9	21.2	28.3	46.1	22.7	46.4	57.5	30.8	65.8	9.5	73.7	35.3	63.2	40.6	29.2
**Secondary+**	23.7	53.4	36.1	21.2	22.2	74.8	48.6	30.1	60.2	19.0	47.7	13.4	42.3	24.0	53.7	69.8
**Wealth tercile**																
**Lowest**	16.8	17.3	15.7	25.8	7.4	27.7	20.3	26.9	33.7	29.6	22.8	23.0	5.4	26.5	19.2	28.1
**Middle**	23.0	30.7	14.4	24.8	13.3	41.9	31.9	27.8	43.5	27.6	20.1	25.0	26.6	28.7	29.8	28.0
**Highest**	60.2	52.0	70.0	49.4	79.2	30.5	47.8	45.3	22.8	42.8	57.1	52.0	68.0	44.8	51.0	43.9
**Lifetime number of sexual partners**																
**0**	6.8	2.4	8.8	0.0	3.2	2.0	2.6	2.6	1.7	4.3	5.5	3.2	0.4	3.8	5.0	4.7
**1**	33.2	9.8	37.9	13.6	26.7	2.3	15.3	16.1	11.6	28.7	15.8	12.5	9.5	14.2	16.4	22.8
**2–3**	47.2	25.4	34.0	38.5	51.1	13.0	39.4	50.1	44.3	49.6	32.2	44.6	52.5	36.9	42.2	42.5
**4+**	12.9	57.6	14.9	44.4	16.9	68.3	40.6	30.7	36.8	17.4	34.8	37.8	36.9	40.9	35.3	28.3
**DK/Missing**	0.0	4.7	4.3	3.4	2.0	14.4	2.0	0.5	5.6	0.0	11.6	1.9	0.7	4.3	1.0	1.6
**Total**	100.0	100.0	100.0	100.0	100.0	100.0	100.0	100.0	100.0	100.0	100.0	100.0	100.0	100.0	100.0	100.0
**Number**	148	584	163	317	400	426	1,435	1,266	1,085	365	207	908	216	1,436	3,704	2,229

Note: Percentages may not sum to total due to rounding.

Table 3 shows that the HIV-positive reproductive-age population is predominantly female in every country. Except in Zambia, more than 6 in every 10 reproductive-age adults with HIV are women; in Gabon women comprise nearly three-fourths of the adult HIV-positive population. In 10 of 16 countries the majority of PLHIV live in urban areas. The vast majority of PLHIV age 15–49—more than 90% in most of the countries and more than 95% in a few—are age 20 and older.

The educational attainment of PLHIV is heterogeneous across countries. In 5 of the 16 countries the majority of PLHIV have attained secondary schooling or above, while in 4 of 16 countries the majority of PLHIV have attained primary education only. PLHIV are disproportionately richer than the HIV-negative population, over-representing the top wealth tercile in every country except Gabon and Namibia. In Burkina Faso, Cameroon, Chad, Ethiopia, Sierra Leone, Tanzania, Togo, and Zambia at least half of PLHIV are in the top wealth tercile.

The lifetime number of sexual partners reported by PLHIV varies by country, with only Cameroon, Ethiopia, Gabon, Malawi, and Togo having a clear categorical majority (of 2–3 lifetime partners in Ethiopia, Malawi, and Togo and 4+ lifetime partners in Cameroon and Gabon). In most countries the plurality of PLHIV report 2–3 lifetime sexual partners. One striking aspect about the distribution of lifetime sexual partners shown in Table 3 is the proportion of PLHIV who say they have never had sex, as many as 9% in Chad. While the possibility of misreporting cannot be ruled out, this finding suggests that a number of PLHIV may have contracted HIV through non-sexual modes of transmission—for example, from contaminated injections or through mother-to-child transmission in infancy.

### Predictors of prior testing

Because “estimated knowledge” is a population-wide average, confidence intervals are unknown. Testing in the past 12 months would be rare among people who had previously known their HIV status, and this characteristic, in turn, is unobserved. For these reasons, we focus our analysis on the proportion of PLHIV ever tested. We measure the extent to which having ever been tested and received the results differs by background characteristics of PLHIV described in the previous section. As our focus is on gaps in coverage, we use the most frequently tested group as the reference group for each characteristic.

Table 4 shows the unadjusted odds ratios (OR) of ever testing for HIV among PLHIV by characteristic. Asterisks indicate the level of significance. Overall, the most consistent unadjusted gaps in testing are among adolescents and those reporting zero lifetime sexual partners (significant gaps in 12 of 16 countries). In 9 of 16 countries, HIV-positive men are statistically significantly less likely than HIV-positive women to have ever been tested. Additionally, in 9 of 16 countries there are gaps in testing among the lowest wealth tercile compared to the highest, and among people with no education versus secondary and above. Importantly, in 3 countries adults reporting 4 or more lifetime sexual partners are less likely to have ever been tested than those reporting 2–3 partners.

**Table 4:** Unadjusted socio-demographic and risk factors associated with ever being tested for HIV among PLHIV, by country.

	Burkina Faso 2010	Cameroon 2011	Chad 2014–15	Cote d’Ivoire 2011–12	Ethiopia 2011	Gabon 2012	Lesotho 2014	Malawi 2015–16	Namibia 2013	Rwanda 2014–15	Sierra Leone 2013	Tanzania 2011–12	Togo 2013–14	Uganda 2011	Zambia 2013–14	Zimbabwe 2015
**Sex**																
***Reference group*: *female***																
**Male**	0.93	0.78	0.93	0.91	0.90	0.45*	0.27**	0.43**	0.34**	0.47	0.15**	0.56**	0.60	0.41**	0.36**	0.38**
**Place of residence**																
***Reference group*: *urban***																
**Rural**	0.24*	0.34**	0.17**	0.30**	0.23**	0.60	0.82	0.97	0.75	0.95	0.85	0.80	0.35**	0.66*	0.73**	0.74
**Age group**																
***Reference group*: *30–39***																
**15–19**	-	0.40*	0.07*	0.28	0.22*	0.02**	0.12**	0.13**	0.08**	0.19*	0.81	0.31**	0.25	0.41**	0.13**	0.22**
**20–29**	1.11	0.74	0.61	0.77	0.67	0.43	0.90	0.90	0.64	1.64	1.34	1.72*	0.57	1.20	0.74*	1.26
**40–49**	0.57	0.74	0.56	0.52	0.60	0.44*	1.10	0.69	0.89	3.68*	0.63	0.84	0.73	0.89	0.66**	0.92
**Educational attainment**																
***Reference group*: *Secondary or higher***																
**None**	0.19*	0.17**	0.15**	0.28**	0.33*	1.12	0.22**	0.62	0.57	0.18	0.66	0.46*	0.31**	0.40**	1.17	0.49
**Primary**	0.52	0.47**	1.13	0.28**	0.76	0.60	0.83	0.76	0.56*	0.12*	1.51	0.65	0.93	0.57**	0.85	0.80
**Wealth tercile**																
***Reference group*: *Highest***																
**Lowest**	0.16**	0.17**	0.19*	0.14**	0.27**	0.52	0.57**	1.02	0.49*	0.92	0.67	0.86	0.24**	0.73	0.73*	0.70
**Middle**	0.35	0.47**	0.05**	0.62	0.28**	0.57	1.09	1.45	0.68	2.48	0.46	0.73	0.36**	0.72	0.92	0.64*
**Lifetime number of sexual partners**																
***Reference group*: *2–3***																
**0**	0.14	0.09*	-	-	0.17*	0.01**	0.10**	0.12**	0.06**	0.05**	0.05**	0.27*	-	0.14**	0.11**	0.14**
**1**	0.93	1.03	0.28*	0.92	1.11	0.83	0.81	0.94	0.57	0.58	0.65	0.95	0.44	1.07	0.77	0.82
**4+**	1.51	1.31	1.83	1.11	0.96	1.50	0.73	1.06	0.83	0.45	0.54	0.96	0.60	0.67**	0.69*	0.68*
**DK/Missing**	-	3.55*	3.03	1.32	1.26	0.50	0.57	0.13	0.41	-	0.08**	2.67	-	0.60	0.50	0.50
**Number**	148	584	163	317	400	426	1,435	1,266	1,085	365	207	908	216	1,436	3,704	2,229

Note

** = p < .01

* = p < .05.

- = insufficient number of cases to produce an estimate.

Table 5 shows adjusted odds ratios (AORs) of HIV testing by background characteristics. Within countries, the number of adjusted gaps in testing that are significant range from one in Burkina Faso and Togo to five in Cameroon, Uganda, and Zambia. Across countries, having no education is the most consistently significant adjusted gap in ever testing (median AOR = 0.31, AOR range 0.03–1.44). Men (median AOR = 0.38, AOR range 0.15–1.11) have significant adjusted gaps in testing in 10 of 16 countries, and persons reporting never having had sex (median AOR = 0.24, AOR range 0.04–0.79) have a significant gap in 7 of 16 countries. Being an adolescent (median AOR = 0.32, AOR range 0.03–0.88) and having a primary education (median AOR = 0.60, AOR range 0.04–1.35) each have significant gaps in 6 of 16 countries.

**Table 5:** Adjusted socio-demographic and risk factors associated with ever being tested for HIV among PLHIV, by country.

	Burkina Faso 2010	Cameroon 2011	Chad 2014–15	Cote d’Ivoire 2011–12	Ethiopia 2011	Gabon 2012	Lesotho 2014	Malawi 2015–16	Namibia 2013	Rwanda 2014–15	Sierra Leone 2013	Tanzania 2011–12	Togo 2013–14	Uganda 2011	Zambia 2013–14	Zimbabwe 2015
**Sex**																
***Reference group*: *female***																
**Male**	1.11	0.60*	0.36	0.73	0.75	0.38*	0.26**	0.27**	0.30**	0.38	0.15**	0.52**	0.58	0.37**	0.33**	0.36**
**Place of residence**																
***Reference group*: *urban***																
**Rural**	0.36	0.75	0.48	0.98	0.33*	0.68	0.98	0.98	1.00	1.03	4.07	0.92	0.59	0.91	0.78	1.62
**Age group**																
***Reference group*: *30–39***																
**15–19**	-	0.64	0.24	0.32	0.88	0.03**	0.12**	0.15**	0.12**	0.43	0.55	0.38	0.30	0.50*	0.21**	0.57
**20–29**	0.61	0.74	0.63	0.87	0.75	0.33*	0.71	0.79	0.61	1.48	0.77	1.53	0.59	0.88	0.75	1.33
**40–49**	0.62	0.81	0.71	0.47*	0.60	0.50	1.21	0.79	1.07	3.32	0.56	0.80	1.07	1.01	0.73*	1.09
**Educational attainment**																
***Reference group*: *Secondary or higher***																
**None**	0.18*	0.24**	0.21*	0.28**	0.44	1.44	0.35**	0.32**	0.57	0.03*	0.41*	0.29**	0.26*	0.24**	0.80	0.38
**Primary**	0.46	0.60*	1.35	0.24**	0.74	0.56	0.86	0.58	0.59	0.04*	0.84	0.50*	0.91	0.47**	0.70*	0.77
**Wealth tercile**																
***Reference group*: *Highest***																
**Lowest**	0.53	0.32**	0.26	0.16**	0.92	0.49	0.80	1.29	0.55	0.62	0.24	1.11	0.53	0.98	0.90	0.50
**Middle**	1.30	0.67	0.07*	0.75	0.75	0.52	1.23	1.91*	0.80	3.28	0.14*	0.91	0.82	0.96	1.04	0.48
**Lifetime number of sexual partners**																
***Reference group*: *2–3***																
**0**	0.15	0.06*	-	-	0.12**	0.05	0.79	0.49	0.31	0.04**	0.06*	0.42	-	0.24**	0.33**	0.27**
**1**	1.28	1.16	0.22**	0.94	0.91	0.70	0.85	0.93	0.71	0.55	0.64	0.86	0.60	1.06	0.82	0.69
**4+**	0.98	1.17	1.72	1.19	1.09	1.35	1.25	1.71	1.20	0.56	0.79	1.22	0.60	0.84	1.06	1.08
**DK/Missing**	-	1.86	4.26	1.54	1.62	0.63	1.09	0.25	0.56	-	0.20	3.99	-	0.97	0.58	0.83
**Number**	148	584	163	317	400	426	1,435	1,266	1,085	365	207	908	216	1,436	3,704	2,229

Note

** = p < .01

* = p < .05.

- = insufficient number of cases to produce an estimate.

## Discussion

HIV testing is an essential gateway to treatment, care, and—through provision of ART and counseling on avoiding risky sexual behavior—a key part of preventing transmission. In its 90-90-90 targets, UNAIDS has called for 90% of PLHIV to know their status, 90% of those who know their status to receive ART, and 90% of ART recipients to achieve viral suppression by 2020. This study has examined factors related to the first of these three targets—HIV testing uptake and estimated knowledge of their HIV status among PLHIV—focusing on 16 countries in sub-Saharan Africa, the region of the world hardest hit by the HIV/AIDS pandemic.

We examined the following six key covariates in relationship to having ever been tested: sex, place of residence, age group, educational attainment, wealth tercile, and lifetime number of sexual partners. Results show that, despite the high risk of HIV that adolescents face, their odds of testing tend to be the lowest of any age group. This is a particularly important challenge because of the high rate of new infections among this group and the broad opportunity for HIV prevention during this critical period in life. In many countries, adolescents face barriers such laws restricting access to testing or requiring parental consent [13, 21] and a particularly strong fear of disclosure [22]. Additionally, laws that criminalize HIV transmission or are used to prosecute PLHIV may have a differentially high deterrent effect on adolescents [13]. Addressing the adolescent gap in HIV testing may require revising national legal frameworks surrounding HIV. Additionally, increasing community-based approaches to testing, integrating HIV testing with other health services such as reproductive health services, and building differentiated service models would likely increase testing among adolescents [13, 23].

We also find important gaps in coverage among men, who are necessary partners in combatting the spread of HIV. Women’s contact with the health system during pregnancy and birth and the increased efforts to test pregnant women likely play a role in this difference in testing. To succeed at halting the epidemic, programs must improve outreach efforts to men and couples. Inviting men to participate in ANC increased their uptake of HIV testing [24, 25], however only a relatively small proportion of partners accepted the invitation. The distribution of self-testing kits to pregnant and post-partum women has been shown to double the uptake of HIV testing among their male partners [26]. Several studies in sub-Saharan Africa have also found that home-based testing has high acceptance rates, and that men are just as likely as women to accept being tested and counseled at home [27]. Taken together, these findings suggest that involving men in ANC, distributing self-testing kits, and expanding home-based testing could substantially increase testing among men. Additionally, male circumcision programs do not routinely offer HIV testing; integrating testing and counseling into these programs would increase testing uptake among men and, by involving their partners in the testing process, could also be an opportune way to further increase testing among women [28].

Additionally, we find gaps in testing among the least educated, and the under-coverage of PLHIV who engage in higher-risk behaviors, such as four or more sexual partners. These findings highlight the particular importance of attracting vulnerable and at-risk populations into testing, perhaps through targeted outreach or using strategies outlined above: integrating HIV testing into routine healthcare services, building differentiated service models, and expanding home-based and self-testing.

### Strengths and limitations

The study made use of comparable, high-quality, recent, nationally-representative data from 16 countries in sub-Saharan Africa. These data enable a population-based analysis of estimated knowledge of HIV status among PLHIV by several important background characteristics. However, the analysis has a few caveats. First, while DHS and AIS survey data are of high quality, some minor variation exists between survey rounds, as discussed earlier. The survey question on having been tested for HIV differs slightly; in early surveys, women and men were asked if they had ever been tested for the “AIDS virus,” but in more recent surveys they are asked if they have ever been tested for “HIV.” In two surveys—Namibia and Uganda—the question about HIV testing is not preceded with the phrase “*I don’t want to know the results*”; PLHIV in those countries who did not want to reveal their status may have been more inclined to state that they had never been tested.

Second, national HIV testing and treatment programs are changing rapidly. Estimates from earlier surveys may not be fully representative of the situation today. Third, supplemental data on ART coverage from UNAIDS is useful for estimating the population of PLHIV who know their status, but it is drawn from data sources that may not align perfectly with a nationally representative household survey. In particular, our estimates of ever tested and recently tested are limited to adults age 15–49, while ART coverage is measured among adults age 15 and older; estimates of coverage may be different among adults over age 50.

Fourth, estimates of knowledge of status should be interpreted with caution for two reasons: first, because the bounds themselves contain uncertainty due to response rates, sampling error, and accuracy of administrative records, and second because the midpoint may not be the best choice to estimate knowledge. It would be preferable to know individual’s exact time since last test and—based on incidence—model the probability of having seroconverted since the last test. Unfortunately, respondents are not asked the month and year of their test unless it was in the past two years, and in some surveys are not even asked the date of test, only whether it was in the past 12 or 24 months. Estimated HIV incidence during the year of survey fieldwork was less than 1% in all countries except Lesotho [19], hence incidence-based adjustments for the upper bound would be unlikely to affect the magnitude of our estimates.

Fifth, while population surveys have become important sources of HIV prevalence estimates, it is important to note that these estimates may include biases resulting from absence or refusal to participate or testing error resulting in false positivity. In general, the higher the non-response, the greater the likelihood that the survey data may be inaccurate. Separating non-response resulting from absence and refusal is important in analyzing the effects of non-response on biomarker estimates. The 2015 UNAIDS/WHO guidelines on monitoring HIV impact using population-based surveys states that a non-response rate greater than 25% is considered high, and the collected characteristics that may be related to non-response should be further assessed, with all related calculations included in the final survey report [29]. As Table 1 showed, the consent rate for HIV testing ranged from 88.7% in Cote d’Ivoire to 99.6% in Rwanda; results should thus be interpreted with caution but are not close to the UNAIDS/WHO level said to be of concern. Yet, to the extent that PLHIV who know their status may be more likely to refuse participation in testing altogether the results may underestimate the proportion of PLHIV who know their status. Additionally, an analysis of detailed lab results from 20 DHS and AIS surveys by Fishel and Garrett [30] indicates that it is likely that testing error associated with false positivity on the ELISA tests in the HIV testing algorithm is present to some degree in many of the surveys analyzed. The magnitude of bias associated with testing error could not be measured by this analysis; however, in many surveys, this bias is likely to fall within the bounds of the confidence interval for the HIV prevalence estimate.

While refusal to be tested may be more common among people who have previously tested positive for HIV, the magnitude of refusal bias in HIV prevalence surveys appears to depend on the study protocol. It has been found that bias is greater when post-test counseling and the return of HIV test results is a prerequisite of study participation [31], which it is not currently in DHS and AIS surveys. Overall, population-based surveys, such as DHS and AIS, have been found to provide reliable, nationally representative direct estimates of HIV seroprevalence in countries with generalized epidemics. HIV prevalence data from population-based surveys can be useful in understanding the size and spread of the epidemic and in adjusting estimates from sentinel surveillance [32].

## Conclusion

The 16 countries included in this study had, at the time of survey fieldwork, an adult HIV prevalence ranging from 1.0% (Burkina Faso) to 24.7% (Lesotho). We found that between 28% (Sierra Leone) and 84% (Rwanda) of PLHIV are estimated to know their status. On average, across study countries, 54% of PLHIV are estimated to know their status. This is encouraging progress, particularly among higher-prevalence countries, but on average it is far short of the 90% goal set by UNAIDS and indicates that nearly half of PLHIV in the average study country cannot receive lifesaving treatment because they are unaware that that are HIV-positive.

Progress on testing and treatment of HIV has undoubtedly been achieved in recent years, but several large gaps in coverage still exist in the 16 study countries among adolescents, men, the least educated, as well as under-coverage of PLHIV who engage in higher-risk sexual behaviors, such as four or more lifetime sexual partners. Taken together, these findings suggest that, in addition to efforts to target people at greatest risk of HIV, there is a continued need to target interventions toward men and toward socially vulnerable groups such as adolescents and the least educated.

Underlying differences in medical infrastructure and in levels of funding for HIV programs contribute to differences in coverage and disparities in the uptake of HIV testing. The experience of countries such as Rwanda, Zimbabwe, Malawi, and Namibia which have been particularly successful at scaling up testing across the population, may provide useful insights for other sub-Saharan countries. Expansion of HIV testing, outreach through mobile clinics, home-based and self-testing, broad coverage through outreach campaigns, community-based approaches, and an integration of opportunities to be tested during regular medical care remain important frontiers for reaching HIV-positive persons in sub-Saharan Africa with lifesaving treatment.
